# The factors associated with paediatric medical post-traumatic stress: A systematic review

**DOI:** 10.1177/13591053241272214

**Published:** 2024-09-30

**Authors:** Ilia Marcev, Colm Lannon-Boran, Philip Hyland, Joanna McHugh Power

**Affiliations:** 1 Maynooth University, Ireland; 2 National College of Ireland, Ireland

**Keywords:** children, families, healthcare, hospitalisation, mental health

## Abstract

We examined and synthesised existing literature on factors associated with paediatric medical-related posttraumatic stress among children and their parents. Children experiencing a broad spectrum of medical conditions, diseases and injuries were of interest. A search of relevant literature concerning PMTS in children and their parents, as well as factors associated with PMTS, was conducted using Medline, PubMed and Scopus. Only studies published in English between January 2018 and November 2023 were included. Twelve articles met inclusion criteria. A broad range of correlates of PMTS were identified for children and parents, which were thematically organised into six key areas: hospital practices and environments; the parent-child relationship; parental mental wellbeing; psychological factors; sociodemographic factors; and the physical consequences of the condition. Bearing in mind constraints on causal inference due to the design of the included studies, knowledge of the factors associated with PMTS may enable clinicians to identify at-risk children and parents, with a view to intervention.

Paediatric medical conditions, injuries and diseases can be traumatising and profoundly distressing experiences (Tedstone and Tarrier, 2003). Paediatric medical-related posttraumatic stress (PMTS) occurs when children, adolescents and/or their parents experience symptoms of posttraumatic stress disorder (PTSD) in response to a medical situation or treatment (Bevilacqua et al., 2021). PMTS includes a broad range of traumatic stress responses such as hyperarousal, negative changes in mood and cognitions, avoidance behaviours and re-experiencing the medial-related traumatic event (Kazak et al., 2005). PMTS affects approximately 19% of child patients and between 10% and 38% of their caregivers (Christian-Brandt et al., 2019; Kahana et al., 2006; Pinquart, 2019).

While the physical burdens of a medical condition or injury lay with the child, parents can be extremely distressed by what is happening to their child (Cousino and Hazen, 2013). The fear of relapse, mistreatment, misdiagnosis and death are concerns that parents of children with chronic and acute illnesses deal with over long periods of time, and do not necessarily disappear post-discharge or in remission (Wakefield et al., 2010). Furthermore, it is known that posttraumatic stress symptoms can lead to an increased likelihood of a deterioration in physical health (McFarlane, 2010). It is critical therefore to better understand the factors that are associated with PMTS in children and their parents. Similar to PTSD, PMTS is often accompanied by behaviours that may provoke a fear of or complete avoidance of healthcare settings, healthcare professionals or medical treatments if not attended to (De Castella et al., 2017; Price et al., 2015). As healthcare is important for wellbeing, those with PMTS may present with a higher likelihood of avoiding necessary medical care, potentially leading to further complications.

## Rationale for the current review

PMTS is a significant concern that can severely affect the physical and psychological wellbeing of children and their parents. Several factors associated with PMTS have been identified in studies involving children and parents including hospitalisation, isolation from family, pain arising from a medical condition, and poor quality of life (QoL) for children (Stanzel and Sierau, 2021; Stuber et al., 1997). However, much of this literature has focused on specific conditions or populations such as cancer, epilepsy, burn patients and transplant patients. While valuable, this narrow focus limits our understanding of PMTS across a broader spectrum of medical contexts. Additionally, there is a need for an up-to-date review that covers a wide spectrum of medical conditions and contexts, accounting also for advancements in medical technologies and practices that have occurred over recent years (Lindblad et al., 2017). Knowledge of child and parent profiles most associated with PMTS could provide clinicians and healthcare workers with insight into dyads who may be more vulnerable to PMTS (Andrade and Devlin, 2015). Finally, it is vital to identify negative hospital practices associated with PMTS to implement healthcare policy improvements (Jazieh and Kozlakidis, 2020). It is important to note that the world of healthcare has changed significantly since the Covid-19 pandemic, with hospitals over-burdened, patients being remanded to off-site or online healthcare, and delayed care becoming ever-prominent (Burau et al., 2024). A review of the factors associated with PMTS across a broad range of medical contexts could help identify maladaptive hospital practices, identify families affected PMTS and inform interventions aimed at reducing the incidence of PMTS and the negative health outcomes that accompany it.

Objective: We reviewed the recent literature (January 2018–November 2023) to identify factors associated with PMTS in children and their parents. This timeframe was chosen to summarise the findings of the last 5 years and to focus on the most recent literature to produce the most up-to-date review of research findings relating to PMTS. By focusing on the last 5 years, we aimed to ensure our review prioritised recent studies to minimise the inclusion of outdated or less relevant research. We also underscore the transformations in healthcare practices worldwide following the Coronavirus Pandemic. Our selected publication date range spans both pre- and post-pandemic healthcare environments to capture these transformations comprehensively.

## Methods

### Search strategy

Electronic databases (Scopus; Medline; PubMed) were searched for primary or secondary studies (i.e. those that collected purposive data or those that analysed secondary data) on PMTS and associated factors published between January 2018 and November 2023. The search was last updated on 20 November 2023. Variants of the search terms ‘medical-related posttraumatic stress’, ‘PTSD’, ‘disease’, ‘parents’ and ‘children’ were used (See Supplemental Materials 2). The reference lists of documents selected for review were searched for other sources that might be eligible for review that fit the inclusion criteria of the current review. We mitigated bias in the review design by formulating a specific research question with predefined inclusion/exclusion criteria. A systematic review protocol was registered on the international database PROSPERO (ID = CRD42023480235).

### Inclusion/exclusion criteria

We included any primary or secondary research quantitatively or qualitatively exploring factors associated with posttraumatic stress in children or parents. Factors were defined as any variables (e.g. neuroticism levels), circumstances (e.g. poor communication from healthcare workers) or events (e.g. pain from treatment) identified as being linked or correlated with the occurrence or severity of PMTS in children or parents. Illness was defined as any physical condition affecting paediatric patients, including but not limited to acute injuries, chronic disease and birth or developmental disorders (World Health Organization, 2023). Studies were selected if published in English between January 2018 and November 2023. Grey literature, unpublished manuscripts, studies primarily focusing on adult patients, abuse-related medical injuries and critically low-quality studies were not included for review. Descriptive studies reporting prevalence rates of PMTS alone or studies that explicitly explored the sequelae of PMTS were excluded to focus specifically on factors associated with PMTS. Though the systematic review excluded non-English language articles, we aimed to reduce bias by using multiple databases and by including a broad range of study designs and methodologies, as well as including as broad a range as possible of health exposures.

### Study selection and risk of bias assessment

Search results from Scopus, PubMed and Medline were downloaded to Mendeley to assess their eligibility. All duplicates were removed from the list of retrieved documents. Two reviewers (IM and CLB) read the titles and abstracts of every article independently to screen for relevance to the review aim. After completing the independent title and abstract screenings, both reviewers reconvened to compare results and establish a consensus on the selected studies. One reviewer (IM) read the full texts of the remaining papers, eliminating papers that did not meet the review criteria, and extracting data from those that did. The second reviewer (CLB) completed an independent check of 14.29% (*n* = 2 of a total of 14). Reference lists of included articles were searched and cited articles were then included if relevant to the review aim and if they fell within the timeframe of the review (January 2018–November 2023).

Risk of bias assessments were conducted by IM and CLB. Owing to the range of studies included for review, the AMSTAR-2 (A Measurement Tool to Assess Systematic Reviews 2; Shea et al., 2017) was used to assess the risk of bias in systematic reviews, the ROBINS-I (Risk of Bias In Non-Randomised Studies – Of Interventions) was used for meta-analyses (Sterne et al., 2016) and the MMAT (Mixed Methods Appraisal Tool; Hong et al., 2018) was used on the quantitative and qualitative studies included in the review.

The AMSTAR-2 has 16 items and rates the overall confidence in the results of a systematic review, with reference to its specific weaknesses, rather than producing an overall score that may not fully indicate the potential areas for bias. AMSTAR-2 scores systematic reviews across four domains that reflect the confidence in the results of the review: high (no critical weaknesses or one non-critical weakness), moderate (more than one non-critical weakness), low (one critical weakness with or without a non-critical weakness) and critically low (more than one critical weakness).

The ROBINS-I tool assesses the risk of bias in estimates of an intervention when studies did not employ a randomisation process for intervention allocation. The tool assesses the risk of bias across seven domains at pre-intervention, intervention and post-intervention. The ROBINS-I tool scores studies in terms of overall risk of bias into the following categories: low risk of bias for all domains; moderate risk of bias for all domains; serious risk of bias in at least one domain; and critical risk of bias in at least one domain. Studies can also be categorised as having insufficient information to evaluate risk of bias.

**Figure 1.**
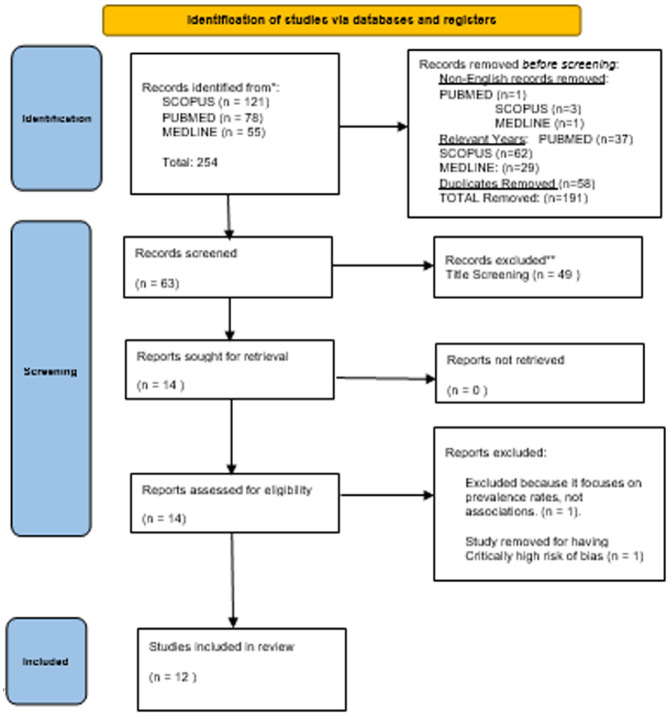
PRISMA flow diagram of identification, screening and inclusion of studies into the current systematic review.

The MMAT assesses the most common types of quantitative, qualitative and mixed-methods studies. The MMAT is used to assess empirical studies based on their design. It assesses qualitative studies based on the data collection methods, participant recruitment, the method of analysis and the rigour and transparency of the interpretation. Quantitative studies are evaluated considering criteria such as the appropriateness of the study design, the randomisation process, data collection methods, adherence to any interventions and the risk of non-response bias. The tool can be used to produce an overall percentage quality criterion met, where 100% indicates that all criteria are met, and 0% indicates that none are met. See Figure 1 for the Preferred Reporting Items for Systematic Reviews and Meta-Analyses (PRISMA) flow diagram (Page et al., 2021).

### Data extraction

Data were extracted by the first author from studies that met the inclusion criteria and did not score critically low for bias in the assessment stage. Data extracted were author name, year of publication, the country the study took place in, the setting where recruitment took place, the condition or injury of the child patient, sample size, mean ages and standard deviations of child participants (where relevant), the measure used and the identified factors associated with PMTS. For quantitative studies and meta-analyses, significance values, effect size scores (Pearson’s *r*, standardised beta values, unstandardised beta values, odds ratios, *t* values, significance values and Cohen’s *d* values) and directions of association were extracted. For qualitative studies and systematic reviews, summaries of findings were extracted. This amalgamation of diverse data sources and extraction techniques aligns with the narrative synthesis methodology employed in the current systematic review, facilitating a nuanced analysis of the identified studies. Two authors were contacted for further data (Colville and Pierce, 2023; Dewan et al., 2022). The RoB assessment for qualitative papers requires congruence between author interpretations of the data and the data itself, and interview transcripts were not accessible in the Dewan et al. (2022) study. Colville and Pierce (2023) did not include the strengths or directions of the associations between factors and PMTS and was contacted for access to this data. Unfortunately, it was not possible to access the data from the two studies. As the risk of bias assessments for both studies indicated a low risk of bias, both studies were included in the current review.

### Data analysis

A narrative synthesis based on the PRISMA reporting guidelines (Page et al., 2021) was used to synthesise, report and interpret the findings from the included studies on PMTS among children and parents. Initially, pertinent information such as study design, participant characteristics, recruitment procedure or setting, and patient illness or condition was extracted and presented in a table (see Table 1). Following data extraction, study findings representing factors associated with PMTS were categorised based on thematic similarity. This categorisation process involved iterative grouping and refinement of study findings, where similar findings were clustered together, while distinct categories were delineated based on patterns and themes that were developed from the data. To ensure consistency and reliability in this process, a constant comparison approach was adopted which involved collaborative discussion between the research team to ensure consensus in the categorisation of study findings. Once complete, we established a final thematic framework consisting of the distinct thematic categories representing the factors associated with PMTS.

**Table 1.** Summary of study findings.

Author and country	Participants	Patient condition	Assessment of PTMS	Factors associated with PMTS
Dewan et al. (2022) – Canada	Mothers (20) Fathers (2) Children (22)	Medical complexity	Parent perspectives from semi-structured interviews	Dismissing parent expertise. Limiting parental autonomy or overburdening them. Lack of trust in HCPs and perceived need for hypervigilance. HCP’s non-trauma-informed language can cause re-traumatisation, and cold communication. Perceived indifference in HCPs and rigid hospital policies that may not be ideal for the wellbeing of the child. Insufficient mental health supports and basic needs.
Colville and Pierce (2023) – UK	Parents (66) Children (66)	Admission to PICU for any reason	Child Revised Impact of Event Scale (CRIES-8), Davidson Trauma Scale (DTS).	Parents: Emergency admission (*p* < 0.001). Parent anxiety levels (*p* = 0.003). Parent depression levels (*p* = 0.001). Parent overprotection of the child (*p* = 0.001). Trajectory of child’s PTS recovery (*p* = 0.018) Children: Emergency admission (*p* = 0.002). Paediatric index of mortality score (*p* = 0.029). Health-related QoL (*p* < 0.001). School-related QoL (*p* < 0.001).
Ari et al. (2018) – Israel	Parents = 88 Children = 88 (mean age = 9.03, SD = 2.3)	Surgeries (urological, orthopaedic, ENT, dermatological, gastroenterology)	UCLA PTSD Reaction Index, Posttraumatic Stress Diagnostic Scale (PSDS)	Child and parent PTSD strongly associated (*r* = 0.417, *p* < 0.001). Hospitalisation reported as most traumatic experience for parents and children. 13.6% of children had a total PTSD score in the clinical range before hospitalisation. This new rose to 27.3% after hospitalisation.
Cuneo et al. (2022) – USA	Children = 132 (mean age = 15, SD ≈ 4.01)	Irritable bowel disease, acute-recurrent/chronic pancreatitis, cystic fibrosis	UCLA PTSD Reaction Index, Impact of Events Scale (IES)	Child PTS severity associated with parental PTS severity (OR = 4.3, *p* < 0.01). Child PTS associated with ICU visits (OR = 4.7, *p* < 0.01). Child PTS associates with high home medication burdens (OR = 3.0, *p* < 0.01). Child PTS associated with government-sponsored or non-sponsored treatment (OR = 2.1, *p* = 0.045)^a^ Child PTS associated with hospitalisation >20 days (OR = 2.8, *p* = 0.01). Child PTS associated with medical comorbidities (OR = 2.0, *p* = 0.04). Only high home medication (*p* = 0.04) and parental PTS (*p* = 0.02) strongly associated with child PTS, controlling for other variables.
Stolz et al. (2021) – USA	Children = 53 (mean age = 16.40, SD = 1.60)	Kidney, liver or heart transplant patients	Child PTSD Symptom Scale (CPSS)	Strong association between neuroticism and PTSS in children (ß = 0.60, *p* < 0.001). 43% of variance in child PTSS explained by neuroticism alone, with executive functioning impairments explaining a further 10%. Children with higher scores in neuroticism are more at risk for PTSS, particularly if they demonstrate higher levels of executive functioning impairments also.
Pinquart (2019) – Germany	184 Studies involving parents (*n* = 21, 224)	Children with asthma, burns, cancer, cleft lip and/or palate, diabetes, epilepsy, food allergy, heart disease, HIV, phenylketonuria, sickle cell disease, spina bifida, other mixed diseases.	IES, PTSD Symptoms Checklist, PDS, PSI, PTSD-C, SCID, PCL-C, Clinician Administered PTSD scale, PCL, TSI, HTQ, TSSS, PPQ, LA Symptom checklist, PTSD-RI, UCLA Index Reaction for DSM-IV, ICD Symptom checklist for mental disorders, Posttraumatic Stress Scale – Interview Version for DSM-5, CIDI, Davidson Trauma Scale, Harvard Trauma Scale, Psychiatrist interviews.	Parental PTSS associated with child illness severity (*r* = 0.18, *p* < 0.001). Parental PTSS associated with treatment duration and intensity (*r* = 0.21, *p* < 0.001). Parental PTSS associated with the recency of final treatment (*r* = −0.10, *p* < 0.001) and recency of diagnosis (*r* = −0.19, *p* < 0.001). Parental PTSS associated with child PTSS (*r* = 0.34, *p* < 0.001). Parental PTSS associated with level of social support (*r* = −0.17, *p* < 0.001) and being married (*r* = −0.07, *p* < 0.001). Parental gender was weakly positively associated with PTSS (*r* = 0.19, *p* < 0.001), where mothers reported higher levels of PTSS than fathers. Parents of children with chronic illnesses considered most at risk.
Beveridge et al. (2018) – Canada	Parents = 204 Children = 173 (mean age = 13.43, SD = 2.53)	Child chronic pain patients	Parents: PTSD Checklist for DSM-V (PCL-5) Children: Child PTSD Symptom Scale (CPSS-V)	Parents: PTS associated with Child PTS (*r* = 0.26, *p* < 0.01). PTS associated with Child HRQoL (*r* = −0.31, *p* < 0.01). PTS associated with child pain interference (*r* = 0.27, *p* < 0.01). Lower income associated with PTS (*r* = −0.22, *p* < 0.01), where lower income was associated with higher PTS levels. Non-white parents have higher PTS than white parents (*t*(182) = −3.09, *p* < 0.01). Children: PTS associated with poorer HRQoL (*r* = −0.55, *p* < 0.01). PTS associated with higher levels of pain interference (*r* = 0.26, *p* < 0.01). PTS weakly linked to lower parental income (*r* = −0.31, *p* < 0.01). PTS associated with younger age (*r* = 0.25, *p* < 0.01).
Eichholz et al. (2023) – Australia	Caregivers = 1908 across 14 studies.	Child chronic pain patients	PTSD Symptom Checklist for DSM-V (PCL-5)	Lower household income associated with higher levels of PTS. Caregivers experiencing chronic pain reported higher PTSS. Caregivers with more stressful life events had higher levels of PTS. Caregivers who catastrophise about their child’s pain had higher levels of PTSS.
Le Brocque et al. (2020) – Australia	Mothers = 272 Children = 272 (mean age = 7.7, SD = 4.52)	Child patients admitted to PICU for more than 8 hours	Total PTS Scale of the Trauma Symptom Checklist for Young Children (TSCYC)	Longer stays in PICU predicted chronic PTSD trajectories (OR = 2.4, *p* < 0.05). Parental perceived threat to life predicted chronic PTSD trajectories (OR = 7.63, *p* < 0.05). Controlling for demographic, parental influence, length of hospital admission, child pre-morbid internalising behaviours^b^ associated with higher levels of PTSD in children (OR = 5.01, *p* < 0.01). Child pre-morbid externalising behaviours^c^ found to be a significant predictor of chronic PTSD trajectories among children (OR = 3.81, *p* < 0.01), but was not a significant predictor when controlling for demographic, length of hospital admission and parental influence
Neville et al. (2018) – Canada	Parents = 102 Children = 102 (mean age = 13.5, SD = 4.2)	Chronic pain patients	Child PTSD Symptom Scale (CPSS), PTSD Checklist for DSM-V (PCL-5)	Significant positive association between child and parents PTSD (*r* = 0.31, *p* < 0.01). Child catastrophising thoughts associated with parent PTSD (*r* = 0.22, *p* < 0.05). Parent catastrophising thoughts associated with parent PTSD (*r* = 0.54, *p* < 0.01). Child catastrophising thoughts associated with child PTSD (*r* = 0.23, *p* < 0.05). Parent catastrophising and child catastrophising positively associated (*r* = 0.36, *p* < 0.01). Child pain interference positively associated with parent PTSD (*r* = 0.22, *p* < 0.001) and child PTSD (*r* = 0.26, *p* < 0.001).
Egberts et al. (2020) – Netherlands/Belgium	Mothers (296) Children (296; mean age = 8.17, SD = 8.70).	Child burn patients	Impact of Event Scale (IES)	Clinically relevant levels of PTS decreased from 49% to 18% among mothers over 18 months, indicating that more mothers report higher levels of PTS closer to the date of their child’s injury. At 1 month post-burn, maternal PTS levels were associated with maternal feelings of fear (*r* = 0.44, *p* < 0.01), sadness (*r* = 0.44, *p* < 0.01), anger (*r* = 0.43, *p* < 0.01), horror (*r* = 0.32, *p* < 0.01), shame (*r* = 0.31, *p* < 0.01) and guilt (*r* = 0.40, *p* < 0.01). At 12 months post-burn, maternal PTS levels were associated with feelings of fear (*r* = 0.40, *p* < 0.01), sadness (*r* = 0.40, *p* < 0.01), anger (*r* = 0.48, *p* < 0.01), horror (*r* = 0.42, *p* < 0.01), shame (*r* = 0.37, *p* < 0.01) and guilt (*r* = 0.37, *p* < 0.01).
Carmassi et al. (2018) – Italy	Parents (138)	Child epilepsy patients	Trauma and Loss Spectrum Self Report (TALS-SR)	Parent gender not associated with PTSD but mothers (43.3%) were more likely to experience partial PTSD than fathers (25.0%). Mothers more likely to report intrusion symptoms (*p* = 0.047), negative alterations in cognitions and mood, alterations in arousal and reactivity.

a Government-sponsored treatment represents any plan or treatment programme partly or fully funded through a governmental body. Non-government-sponsored treatment does not indicate private healthcare treatment, rather treatment from non-profit organisations or entities that operate independently from government funding.

b Pre-morbid internalising behaviours represent a child’s tendency to internalise emotional struggles. They often manifest as anxiety, depression, social withdrawal and dissociation.

c Pre-morbid externalising behaviours represent a child’s tendency to process emotional struggles through aggression, emotional and behavioural impulsivity and hyperactivity.

## Results

### Study characteristics

We identified 254 records from the databases, of which 58 were duplicates. Setting the publication date range and eliminating non-English records, 63 records were left. From these, 49 were removed during the title and abstract screening stage, and 14 records were retained and assessed for eligibility. Of these, two records were removed; one for focusing on prevalence rates of PMTS rather than associated factors (Turgoose et al., 2021), and one for scoring critically low in the RoB assessment stage (Carmassi et al., 2019). Overall, 12 studies were included in the review (Ari et al., 2018; Beveridge et al., 2018; Carmassi et al., 2018; Colville and Pierce, 2023; Cuneo et al., 2022; Dewan et al., 2022; Egberts et al., 2020; Eichholz et al., 2023; Le Brocque et al., 2020; Neville et al., 2018; Pinquart, 2019; Stolz et al., 2021). We excluded 242 articles in total (See Figure 1). Data were reported from nine countries: Canada (3), Italy (1), Belgium (1), The Netherlands (1), Australia (2), the United States of America (2), The United Kingdom (1), Germany (1) and Israel (1).

Study designs included qualitative (Dewan et al., 2022), quantitative descriptive (Ari et al., 2018; Beveridge et al., 2018; Carmassi et al., 2018; Colville and Pierce, 2023; Cuneo et al., 2022; Egberts et al., 2020; Le Brocque et al., 2020; Neville et al., 2018), quantitative non-randomised (Stolz et al., 2021), a systematic review (Eichholz et al., 2023) and a meta-analysis studies (Pinquart, 2019). There was a combined total of 25,524 participants across the 12 studies. Three studies (25%) were assessed as having met 100% quality criteria, seven studies (58.33%) met 80% quality criteria, and one presented low concern of bias (8.03%; Pinquart, 2019). One study presented low quality (Eichholz et al., 2023) due to not conducting a risk of bias assessment. However, this study was included as the authors addressed the potential impact of bias on their findings (See Supplemental Materials 1 for study characteristics).

### Identified themes

#### PMTS and hospital practices/environments

PMTS occurred in the context of the medical care setting, in relation to its policies and practices, and interactions between parents, patients and healthcare professionals. Hospital practices and environments were identified as locations of traumatic experiences for children and parents facing health challenges. The correlates most addressed by the research were hospitalisation and emergency admission (Ari et al., 2018; Colville and Pierce, 2023; Cuneo et al., 2022).

Children admitted by emergency to intensive care units or other wards were significantly more likely to report higher levels of PMTS than children admitted to hospital for non-emergency reasons (Ari et al., 2018; Colville and Pierce, 2023; Cuneo et al., 2022). Additionally, children spending longer periods in paediatric intensive care unit wards were also more likely to report longer lasting PMTS symptoms (Le Brocque et al., 2020). Through qualitative interviews with parents of child patients admitted to ICU, Dewan et al. (2022) reported that parents felt significantly more distressed by the hospital policies in place that they believed were unsuitable for their child’s particular medical context. Parents also reported that poor communication from healthcare providers (HCPs), where health updates were delivered in an indifferent or detached manner in a cold environment, contributed to their feelings of distress (Dewan et al., 2022). Furthermore, parents who felt their expertise regarding their child’s health was being dismissed by their HCPs also experienced feelings of hopelessness and increased stress as a result. Cuneo et al. (2022) also observed that children who were receiving government-sponsored treatment or non-government-sponsored treatment were twice as likely to have higher levels of PMTS than children accessing treatment through private means. Notably, parents with insufficient mental health supports or physical supports such as regular nutritious food, water and rest from their caregiving burdens, report high stress levels (Dewan et al., 2022). Overall, parents and children who spend long periods of time in hospital for emergencies, accessing treatment through non-private healthcare, receiving health updates from HCPs using distressing language and terminology, and parents who are not being adequately supported, are significantly more at risk of developing PMTS.

#### PMTS and parent-child relationship

We identified a consistent theme concerning the family dynamic or relationship between parents and their children and PMTS. Eight studies reported numerous factors within this category (Ari et al., 2018; Beveridge et al., 2018; Colville and Pierce, 2023; Cuneo et al., 2022; Dewan et al., 2022; Le Brocque et al., 2020; Neville et al., 2018; Pinquart, 2019).

The associations between child and parent PTSD levels were assessed across five studies included in the current review. Ari et al. (2018) reported a significant positive relationship between child PTSD and parental PTSD (*r* = 0.417, *p* < 0.01). Overall, significant weak-to-moderate associations were observed between child and parent PTSD levels (Beveridge et al., 2018; Cuneo et al., 2022; Le Brocque et al., 2020; Neville et al., 2018; Pinquart, 2019). Conversely, parents who observed elevated levels of PMTS in their own children, coupled with distress exacerbated by hospital practices and environments, may experience heightened PMTS. As demonstrated by Colville and Pierce (2023), parents with higher levels in hypervigilance over their children are also more likely to report PMTS. As such, parents who feel a need to protect their children from danger may feel a sense of hopelessness if their children have chronic conditions that make treatment difficult. Additionally, where parents perceive a threat to the child’s life, children are more likely to report longer lasting PMTS (OR = 7.63, *p* < 0.05), however this association was non-significant when controlling for other variables. This reciprocal relationship between child and parent PTSD levels may be fuelled by the distress observed by each party, with children affected by their parents’ responses, and parents affected by their children’s responses.

#### PMTS and parental mental wellbeing

This category concerns the relationship between PMTS and co-existing mental health conditions or distress faced by parents. Throughout the review process, we came across two studies (Colville and Pierce, 2023; Eichholz et al., 2023) that analysed the effects of mental wellbeing and distress on PMTS. Notably, our review identified a gap in the existing literature – no studies were identified that examined the mental health of children as a factor associated with PMTS in either children or parents. Parent anxiety and depression levels were found to be associated with PMTS among parents (Colville and Pierce, 2023). Parents who had higher levels of anxiety or depression were more likely to report PMTS than parents with lower levels. Notably, Eichholz et al. (2023) reported that parents with prior experience with stressful life events were more likely to have higher PMTS levels.

#### PMTS and psychological factors

This section examines the psychological dimensions that contribute to the development of PMTS among parents and children. These include parental and child thought patterns, emotional responses, individual differences and cognitive functioning. The specific factors identified under this theme were parental catastrophising thoughts, child catastrophising thoughts, premorbid child internalising and externalising thoughts, basic emotions (fear, anger, sadness, horror), self-conscious emotions (shame, guilt), neuroticism and child impairments in executive functioning.

In terms of personality traits, only neuroticism was found to be associated with PMTS, where children with higher scores in neuroticism were more associated with PMTS (Stolz et al., 2021). Notably, children with executive functioning (EF) impairments, such as working memory, inhibition and problem-solving impairments, were not associated PMTS, but children with high neuroticism and EF impairments were (Stolz et al., 2021). Furthermore, child catastrophising thoughts were observed to be associated with child and parental PTSD (Neville et al., 2018). Parental catastrophising was associated with child/parental PMTS. Child and parent catastrophising thoughts were associated with each other. Thus, children whose parents display PMTS were more likely to catastrophise and vice versa (Eichholz et al., 2023). Dyads where both child and parent tended to catastrophise were more likely to report higher levels of PMTS.

When controlling for demographic, length of hospital admission and parental emotional influence, child pre-morbid internalising behaviours were associated with long-lasting PTSD among children (Le Brocque et al., 2020). In essence, children who tend withdraw, dissociate, are prone to depression or anxiety, or internalise emotional struggles when distressed are particularly associated with long-term PMTS. No evidence was available relevant to parental internalising behaviours, but notable associations between maternal PMTS levels and a range of emotional responses were observed. Among mothers of child burn patients, at 1-month post-burn, maternal PMTS was strongly correlated with heightened feelings of fear, sadness, horror, anger, shame and guilt (Egberts et al., 2020). These associations remained persistent at the 12-month follow-up, indicating that basic and self-conscious emotions are strongly associated with PMTS among mothers.

#### PMTS and socio-demographic factors

Several sociodemographic variables were identified as factors associated with PMTS in the literature, encompassing gender, age, ethnicity, household income, marital status and social support. The association between child and parent socio-demographic was explored in four studies included in the current review. Mothers were found to be more likely to report higher PTSD levels than fathers, but this association was weak (Pinquart, 2019). Conversely, Carmassi et al. (2018) did not observe a significant association between parent gender and PTSD but reported a significant difference between the prevalence of partial PTSD between parents, where mothers were more likely to experience intrusion symptoms, mood alterations and changes in arousal. Pinquart (2019) reported a weak negative association between parent PTSD scores and being married, where married parents tended to report lower PTSD scores. Beveridge et al. (2018) and Eichholz et al. (2023) both observed an association between household income and child and parent PTSD, with child PTSD being more strongly associated with lower household income. The age of the child was also found to associated with PMTS, where younger children tended to report higher levels of PTSD than older children, and parents of younger children also tended to report higher levels of PTSD (Beveridge et al., 2018). Finally, Beveridge et al. (2018) identified an association between parent ethnicity and parent PTSD, where non-white parents experienced higher levels of PTSD.

#### PMTS and physical consequence of condition/disease

The current review included a range of studies that recruited participants across a broad spectrum of chronic and acute diseases, injuries and conditions. While the reviewed articles encompassed diverse medical conditions and illnesses, ranging from acute to chronic, factors associated with PMTS in parents and children were identified across multiple studies, despite the variation in medical contexts. These factors were the health-related quality of life of the child (HRQoL), the Paediatric Index of Mortality (PIM) of the child, high home medication burdens, the presence of medical comorbidities, illness severity, chronic diseases or illness, treatment duration and intensity, the recency of the diagnosis or injury, the recency of the final treatment and child pain interference.

The impact of adverse physical consequences of the child’s condition or disease on PMTS outcomes were explored in six studies included in the current review. The HRQoL of the child was associated with PMTS in both children and parents. A strong negative association between both parent/child PTSD, and child HRQoL was observed, where higher levels of both parent and child PTSD were associated with negative child HRQoL (Beveridge et al., 2018). Notably, Colville and Pierce (2023) reported that poorer child emotional QoL was a factor associated PMTS, with poorer social and school related QoL emerging as correlates also. Thus, children who experience isolation or a negatively impacted social and school related QoL also tend to report higher levels of PMTS. Children with high home medication burdens to treat a chronic disease or illness also reported higher levels of PMTS (Cuneo et al., 2022). Additionally, children with a higher Paediatric Index of Mortality (PIM) score, which reflects the likelihood of death in critically ill children, tended to report higher levels of PMTS than children with lower PIM scores (Colville and Pierce, 2023). Following this, where children reported a greater illness severity, parents were more likely to report higher levels of PMTS (Pinquart, 2019). Parents were also more likely to report PMTS closer to the date of their children’s final treatment, the diagnosis, and with greater treatment intensity or duration (Egberts et al., 2020; Pinquart, 2019). Child pain interference was associated with both child and parental PMTS. Beveridge et al. (2018) observed that child and parent PTS were equally associated with increased levels of child pain interference. Neville et al. (2018) reported similar results, with child PMTS more strongly associated with child pain interference than parent PMTS.

## Discussion

The current review synthesised the factors associated with PMTS in children and their parents across a broad range of medical issues and conditions. Through systematic analysis of the recent literature, several factors were identified across different countries, medical contexts, healthcare settings and patient/parent demographic. By grouping the factors into themes, we identified six key areas associated with PMTS: hospital practices and environments; the parent-child relationship; parental mental wellbeing; psychological factors; sociodemographic factors; and the physical consequences of the condition or disease.

The hospital environment and its practices were consistently identified across the literature as being associated with child and parental PMTS. Firstly, the emergency nature of hospital admissions for child patients, particularly to intensive care units, was a significant correlate of PMTS in children and their parents. Prolonged stays in paediatric intensive care units contributed to longer-lasting PMTS in children. This highlights the critical importance of considering the impact of hospitalisation and emergency care on patients’ wellbeing. However, past literature suggests that over a third of medical professionals do not believe hospital environments to be stressful on patients (Al-Yateem et al., 2015). Overall, parental attitudes regarding rigid hospital policies, detached or dismissive communication from healthcare professionals, and lack of support from healthcare workers provides evidence to suggest hospitals are lacking flexibility in delivering tailored care to patients. This is further evidenced in past studies involving child patients and caregivers, where families who spent more days in hospital had significantly higher levels of distress than a non-hospitalised control group (Commodari, 2010; Lerwick, 2016).

Interestingly, Commodari (2010) demonstrated that child participation in recreational activities within the hospital environment such as play did not significantly decrease levels of stress in children and caregivers, but participation in scholastic learning delivered by a tutor aided in reducing feelings of anxiety and irritability. Evidence does suggest play can buffer against distress among hospitalised children, however only where play is guided by a trained professional such as a play therapist (Koukourikos et al., 2015; Potasz et al., 2012). This might also suggest a social component involved in child patients’ experience of PMTS. Children who spend long periods in hospital may miss the social support networks they typically have at school (Lerwick, 2016; McLaughlan, 2017). Gariépy and Howe (2003) conducted a study on child patients and found that their tendency to play was significantly lower than healthy children, their levels of anxiety were a primary predictor of non-play, and that when engaging in group play activities their mood tended to improve. As demonstrated in the current review, hospitalised children with poorer school-related QoL were more likely to have PMTS. Therefore, there is reason to suggest that providing distractions in the form of play or toys to children may not be adequate as the primary intervention to foster mental well-being. As children spend a large proportion of their time in school, it may instead prove beneficial to their sense of security and identity to supply tutors that visit the hospital and teach the children.

Notably, the mode of treatment accessibility also appears to plays a role in PMTS, where children receiving government-sponsored or non-government sponsored treatment were twice more likely to exhibit higher levels of PMTS than children accessing private healthcare. This might be attributed to a higher quality of hospitality and promptness of care often available in private healthcare; a past review has indicated that while the quality of care was not significantly different between public and private healthcare systems, private healthcare tended to deliver healthcare more promptly (Basu et al., 2012). As such, associations between private healthcare environments and PMTS may be weaker than with public or sponsored healthcare, but future research could investigate this.

Children who undergo higher home medication burdens for chronic diseases tended to report higher levels of PMTS. While more serious conditions that require these burdens may also present greater physical symptom severity and may incur more stress, recent evidence suggests that children experience stigma when using medication (Ahmad et al., 2020; Kansra et al., 2021). Children feel different from their peers, especially when using treatment delivery apparatuses such as inhalers or injections. Social exclusion from their peers, who may be uneducated on the nature of their condition, is also a common experience children with chronic illnesses face, which can be exacerbated by severe or frightening physical symptoms (Iovino et al., 2023). While reflective of a wider societal issue and a lack of public knowledge of the lived experiences of child patients, hospitals or local communities could provide or facilitate peer support networks to child patients to foster a sense of belonging, simultaneously help reduce stigma, and perhaps the incidence of PMTS.

The current review identified hypervigilance and catastrophising in parents as a correlate of parent PMTS. Children were also more likely to have PMTS if their parents also had PMTS. Possible explanations for these associations might be children feeling elevated levels of distress when observing the PMTS responses of their parents. Children rely on their parents during times of crisis or distress (Zatzick et al., 2011). Where parents present high levels of hypervigilance, thus spending longer periods of time tending to their children, their children may feel an increased level of worry or distress. Additionally, where parents perceived a threat to the child’s life, they and their children are more likely to report longer-lasting PMTS. A recent study observed that educating parents on their children’s painful medical condition reduced parental catastrophising and hypervigilance significantly after only 1 week of classes (Pintó et al., 2021). Parents in other studies have consistently outlined a need for guidance and education from their children’s healthcare team as vital to better understand the magnitude of their child’s condition and the required level of parental care (Al-Yateem et al., 2015; Tsironi and Koulierakis, 2017). Healthcare workers should therefore aim for an increased level of transparency in delivering healthcare. Doing so could help alleviate stress on parents and enable them to better support their children. Considering both children and their parents with higher scores in neuroticism were more likely to have PMTS, educating both parents and children about their medical condition and treatment options could be particularly beneficial in reducing worry and anxiety.

Both depression and anxiety levels were found to be significantly associated with PMTS among parents, where parents with higher anxiety and depression scores tended to also report higher levels of PMTS. In past research, Santacroce (2002) identified a strong positive relationship between levels of anxiety in parents of child cancer patients and their PMTS levels. Additionally, depression has been found to be strongly associated with PMTS responses such as intrusion, avoidance and arousal among parents of child parents (Norberg and Boman, 2008). Therefore, parents who report higher levels of anxiety and depression are associated with a greater likelihood of experiencing PMTS. Simple quick screenings of parental depression and anxiety when their child is admitted into care might help clinicians identify parents who might present an increased likelihood of additional mental health supports.

With regards to gender differences among parents in the prevalence of PMTS, results were mixed. Reviewed studies either found small positive associations between mothers and PMTS, or no differences in PMTS severity but rather differences in the display of PMTS responses. Mothers were more likely to report intrusion symptoms, mood alterations and changes in arousal. According to the literature, females are at a higher risk than males for developing PTS following a trauma, possibly because of sex differences in physiological responses to traumatic events (Irish et al., 2011). Clinicians could remain aware that depending on medical contexts and parents’ history with traumatic events, mothers may be somewhat more likely to experience PMTS, or at the very least display PMTS responses differently to fathers. As such, mothers may require more tailored supports designed to alleviate their unique PMTS responses.

Household income was negatively correlated with PMTS in children and parents, with higher income being associated with lower levels of PMTS. From world mental health surveys, a significant difference has been observed between the rates of PTSD treatment-seeking in higher-income countries (53.5%) and low-lower middle income (22.8%) countries (Koenen et al., 2017). Thus, parents with higher incomes may be more likely to afford and seek psychological treatment for their own and their children’s PMTS. Similarly, they may also be more likely to afford the promptness of treatment associated with private healthcare. To better support parents and children from lower-income households, government funding towards public healthcare systems may need to increase significantly.

The age of the child presented as a correlate of PMTS, where younger children reported higher levels of PMTS than older children, and parents of younger children also reported higher levels of PMTS. Notably, research suggests that maladaptive coping mechanisms such as aggression and internalising behaviours are more associated with younger child ages (Hampel and Petermann, 2005). As such, younger children who reported higher levels of PMTS may be utilising maladaptive coping mechanisms to process the emotional distress they experience in their medical contexts. Aggressive behaviours in particular might be distressing on parents to observe and manage, and place a strain on a parent’s capacity for self-care practices to help mitigate their distress. Notably, research suggests that children with aggressive behaviours and poor coping skills also tend to have parents with similar problems, such as high irritability, and inadequate emotion regulation skills (Krahé et al., 2014). Short-term therapeutic interventions such as mindfulness for children to help equip them with the skills to regulate their stress may also be beneficial for their parents (Wenzel et al., 2020).

A weak correlation between parent ethnicity and parent PMTS was noted in the current review, where non-white parents experienced higher levels of PMTS than white parents. The research on ethnicity and PTSD prevalence is mixed, but some studies suggest that discrimination, stigma and specific ethnicity may mediate the relationship (Asnaani and Hall-Clark, 2017). Consequently, in the medical context, stigma associated with healthcare delivery could be a barrier to accessibility of services, which could exacerbate feelings of distress among non-white ethnicity families.

### Limitations

This review is limited in its capacity to infer causality – we can only report factors associated with PMTS among children and parents and cannot infer directional relationships. Additionally, with evolutions in medical practice and procedures, the review will require future updates to capture emerging trends and newly identified factors associated with PMTS. Continuous updates to this review could provide paediatric clinicians with relevant information to improve preventive strategies and interventions for PMTS in paediatric healthcare. Owing to a lack of resources and multilingual personnel, the current review focused solely on English language studies. As a result, the current review may have overlooked relevant literature pertaining to factors associated with PMTS. Furthermore, access to additional relevant data from certain authors was not feasible in order to safeguard participant privacy. Through narratively synthesising our data, the themes presented are subjective and depend on the interpretation of the authors. By excluding grey literature from the current review, we may have inadvertently excluded some studies or reports that could have provided further insights into the factors associated with PMTS.

### Implications for practitioners and future research

The current review demonstrated a gap in the literature that future research could investigate; no recent evidence was retrieved exploring the relationship between child mental health and PMTS in children or parents. Future research could employ longitudinal studies to examine the impact of PMTS on the psychological wellbeing of children and parents over time. This could lead to an understanding of the long-term effects of PMTS beyond childhood. More immediate solutions were proposed. Healthcare professionals should aim for a greater level of transparency in treatment delivery, aiming for a more holistic approach to child patient healthcare. Children facing long-term hospitalisation could benefit from guided group play therapy and a continuation of their education via tutoring. Mental health screenings of parents are of vital importance to help identify parents who might be more likely to develop PMTS, particularly in public hospitals where the prevalence of PMTS tend to be higher. Additionally, healthcare services could work alongside patient advocacy groups to facilitate peer support groups for children in hospitals to reduce the feelings of stigma they face. Overall, an increase in mental health, physical health and emotional supports for parents are needed.

## Conclusion

This review highlights several key factors associated with PMTS in children and their parents. Hospital practices, healthcare workers’ communication, the hospital environments themselves and prolonged stays in intensive care units were identified as correlates of PMTS. Children with high medication burdens may benefit from involvement in peer support groups. Younger children appear to more prone to PMTS, necessitating early interventions and support mechanism to address their emotional needs. Non-white families tended to experience PMTS more than white ethnicity families, perhaps indicative of barriers in healthcare accessibility. While mixed, studies suggest mothers may be somewhat more susceptible to PMTS, or demonstrate different trauma responses to fathers, highlighting a need for tailored support systems for mothers. Future research could employ longitudinal studies to understand the long-term effects of PMTS. Overall, there is an immediate need for increased mental health, physical health and emotional support for parents and children facing PMTS.

## Supplemental Material

sj-docx-1-hpq-10.1177_13591053241272214 – Supplemental material for The factors associated with paediatric medical post-traumatic stress: A systematic review Supplemental material, sj-docx-1-hpq-10.1177_13591053241272214 for The factors associated with paediatric medical post-traumatic stress: A systematic review by Ilia Marcev, Colm Lannon-Boran, Philip Hyland and Joanna McHugh Power in Journal of Health Psychology

sj-docx-2-hpq-10.1177_13591053241272214 – Supplemental material for The factors associated with paediatric medical post-traumatic stress: A systematic review Supplemental material, sj-docx-2-hpq-10.1177_13591053241272214 for The factors associated with paediatric medical post-traumatic stress: A systematic review by Ilia Marcev, Colm Lannon-Boran, Philip Hyland and Joanna McHugh Power in Journal of Health Psychology

sj-docx-3-hpq-10.1177_13591053241272214 – Supplemental material for The factors associated with paediatric medical post-traumatic stress: A systematic review Supplemental material, sj-docx-3-hpq-10.1177_13591053241272214 for The factors associated with paediatric medical post-traumatic stress: A systematic review by Ilia Marcev, Colm Lannon-Boran, Philip Hyland and Joanna McHugh Power in Journal of Health Psychology
