# Supplementary material for: The factors associated with paediatric medical post-traumatic stress: A systematic review
Source: J Health Psychol. 2024 Sep 30;30(11):2860–80. doi: 10.1177/13591053241272214 (PMC12433540; doi:10.1177/13591053241272214)
Supplement: sj-docx-1-hpq-10.1177_13591053241272214 – Supplemental material for The factors associated with paediatric medical post-traumatic stress: A systematic review [file sj-docx-1-hpq-10.1177_13591053241272214.docx]

**Risk of Bias Assessments MMAT**

Dewan et al. 2023

|  | YES | NO | CANT TELL | COMMENTS |
| --- | --- | --- | --- | --- |
| **Are there clear research questions?** | X |  |  |  |
| **Do the collected data allow to address the research questions?** | X |  |  |  |
| Is the qualitative approach appropriate to answer these questions? | X |  |  |  |
| Are the qualitative data collection methods adequate to address the research question? | X |  |  |  |
| Are the findings adequately derived from the data? | X |  |  |  |
| Is the interpretation of results sufficiently substantiated by data? |  |  | X | Access to interview transcripts was not possible as participant information could be compromised. |
| Is there coherence between qualitative data sources, collection, analysis and interpretation? | X |  |  |  |

Collville et al. 2023.

|  | YES | NO | CANT TELL | COMMENTS |
| --- | --- | --- | --- | --- |
| **Are there clear research questions?** | X |  |  |  |
| **Do the collected data allow to address the research questions?** | X |  |  |  |
| Is the sampling strategy relevant to address the research question? | X |  |  |  |
| Is the sample representative of the target population? | X |  |  |  |
| Are the measurements appropriate? | X |  |  |  |
| Is the risk of nonresponse bias low? |  | X |  | 102 families provided data at 3 months (a response rate of 77%) and 72 families provided data at 12 months. The drop in the number of families providing data from 3 to 12 months (from 102 to 72) is also important to note. No information provided on the characteristics of the excluded people. |
| Is the statistical analysis appropriate to answer the research question? | X |  |  |  |

**Ben Ari et al. 2019.**

|  | YES | NO | CANT TELL | COMMENTS |
| --- | --- | --- | --- | --- |
| **Are there clear research questions?** | X |  |  |  |
| **Do the collected data allow to address the research questions?** | X |  |  |  |
| Is the sampling strategy relevant to address the research question? | X |  |  |  |
| Is the sample representative of the target population? | X |  |  |  |
| Are the measurements appropriate? | X |  |  |  |
| Is the risk of nonresponse bias low? |  |  | X | No information provided on nonresponse bias. The information provided mentions that 43 parents refused to participate, but it doesn't offer details about the characteristics of this group.  However, the passage does mention the following:  "There were no significant differences between the demographic characteristics of this group and those of the study sample." "Moreover, there were no differences in the frequency of the type of surgery and in the cause of hospitalization (emergency or elective)." These statements suggest that the group of parents who refused to participate did not significantly differ from the study sample in terms of demographic characteristics, type of surgery, and cause of hospitalization. Need more detail on the rate of response. |
| Is the statistical analysis appropriate to answer the research question? | X |  |  |  |

**Cuneo et al., 2022.**

|  | YES | NO | CANT TELL | COMMENTS |
| --- | --- | --- | --- | --- |
| **Are there clear research questions?** | X |  |  |  |
| **Do the collected data allow to address the research questions?** | X |  |  |  |
| Is the sampling strategy relevant to address the research question? | X |  |  |  |
| Is the sample representative of the target population? | X |  |  |  |
| Are the measurements appropriate? | X |  |  |  |
| Is the risk of nonresponse bias low? |  |  | X | No information provided on nonresponse bias. No information provided on any missing data. 132 participants were recruited and 132 provided data. Paper reads like there was no missing data or nonresponders but needs clarification. |
| Is the statistical analysis appropriate to answer the research question? | X |  |  |  |

**Stolz et al. 2021.**

|  | YES | NO | CANT TELL | COMMENTS |
| --- | --- | --- | --- | --- |
| **Are there clear research questions?** | X |  |  |  |
| **Do the collected data allow to address the research questions?** | X |  |  |  |
| Are the participants representative of the target population? | X |  |  |  |
| Are measurements appropriate regarding both the outcome and intervention (or exposure)? | X |  |  |  |
| Are there complete outcome data? | X |  |  |  |
| Are the confounders accounted for in the design and analysis? | X |  |  |  |
| During the study period, is the intervention administered (or exposure occurred) as intended? | X |  |  |  |

**Pinquart, 2019.**

| **Study Eligibility** | YES | PROB YES | PROB NO | NO | NO INFO |
| --- | --- | --- | --- | --- | --- |
| Did the review adhere to pre-defined objectives and eligibility criteria? | X |  |  |  |  |
| Were the eligibility criteria appropriate for the review question? | X |  |  |  |  |
| Were eligibility criteria unambiguous? | X |  |  |  |  |
| Were any restrictions in eligibility criteria based on study characteristics appropriate (e.g. date, sample size, study quality, outcomes measured)? | X |  |  |  |  |
| Were any restrictions in eligibility criteria based on sources of information appropriate (e.g. publication status or format, language, availability of data) | X |  |  |  |  |
|  |  |  |  |  |  |
| **IDENTIFICATION AND SELECTION OF STUDIES** | YES | PROB YES | PROB NO | NO | NO INFO |
| Did the search include an appropriate range of databases/electronic sources for published and unpublished reports? | X |  |  |  |  |
| Were methods additional to database searching used to identify relevant reports? |  |  |  | X |  |
| Were the terms and structure of the search strategy likely to retrieve as many eligible studies as possible? |  | X |  |  |  |
| Were restrictions based on date, publication format, or language appropriate? | X |  |  |  |  |
| Were efforts made to minimise error in selection of studies? | X |  |  |  |  |
|  |  |  |  |  |  |
| **DATA COLLECTION AND STUDY APPRAISAL** | YES | PROB YES | PROB NO | NO | NO INFORMATION |
| Were efforts made to minimise error in data collection? |  | X |  |  |  |
| Were sufficient study characteristics available for both review authors and readers to be able to interpret the results? | X |  |  |  |  |
| Were all relevant study results collected for use in the synthesis? | X |  |  |  |  |
| Was risk of bias (or methodological quality) formally assessed using appropriate criteria? | X |  |  |  |  |
| Were efforts made to minimise error in risk of bias assessment? |  | X |  |  |  |
|  |  |  |  |  |  |
| **SYNTHESIS AND FINDINGS** | YES | PROB YES | PROB NO | NO | NO INFO |
| Did the synthesis include all studies that it should? | X |  |  |  |  |
| Were all pre-defined analyses reported or departures explained? | X |  |  |  |  |
| Was the synthesis appropriate given the nature and similarity in the research questions, study designs and outcomes across included studies? | X |  |  |  |  |
| Was between-study variation (heterogeneity) minimal or addressed in the synthesis? | X |  |  |  |  |
| Were the findings robust, e.g. as demonstrated through funnel plot or sensitivity analyses? | X |  |  |  |  |
| Were biases in primary studies minimal or addressed in the synthesis? |  | X |  |  |  |
|  |  |  |  |  |  |
| **RISK OF BIAS IN THE REVIEW** | YES | PROB YES | PROB NO | NO | NO INFO |
| Did the interpretation of findings address all of the concerns identified in Domains 1 to 4? | X |  |  |  |  |
| Was the relevance of identified studies to the review's research question appropriately considered? | X |  |  |  |  |
| Did the reviewers avoid emphasizing results on the basis of their statistical significance? | X |  |  |  |  |

**Beveridge et al. 2018.**

|  | YES | NO | CANT TELL | COMMENTS |
| --- | --- | --- | --- | --- |
| **Are there clear research questions?** | X |  |  |  |
| **Do the collected data allow to address the research questions?** | X |  |  |  |
| Is the sampling strategy relevant to address the research question? | X |  |  |  |
| Is the sample representative of the target population? | X |  |  |  |
| Are the measurements appropriate? | X |  |  |  |
| Is the risk of nonresponse bias low? | X |  |  |  |
| Is the statistical analysis appropriate to answer the research question? | X |  |  |  |

**EICHOLZ et al. 2023.**

| **Study Eligibility** | YES | PROB YES | PROB NO | NO | NO INFO |
| --- | --- | --- | --- | --- | --- |
| Did the review adhere to pre-defined objectives and eligibility criteria? | X |  |  |  |  |
| Were the eligibility criteria appropriate for the review question? | X |  |  |  |  |
| Were eligibility criteria unambiguous? | X |  |  |  |  |
| Were any restrictions in eligibility criteria based on study characteristics appropriate (e.g. date, sample size, study quality, outcomes measured)? | X |  |  |  |  |
| Were any restrictions in eligibility criteria based on sources of information appropriate (e.g. publication status or format, language, availability of data) | X |  |  |  |  |
|  |  |  |  |  |  |
| **IDENTIFICATION AND SELECTION OF STUDIES** | YES | PROB YES | PROB NO | NO | NO INFO |
| Did the search include an appropriate range of databases/electronic sources for published and unpublished reports? | X |  |  |  |  |
| Were methods additional to database searching used to identify relevant reports? | X |  |  |  |  |
| Were the terms and structure of the search strategy likely to retrieve as many eligible studies as possible? |  | X |  |  |  |
| Were restrictions based on date, publication format, or language appropriate? | X |  |  |  |  |
| Were efforts made to minimise error in selection of studies? | X |  |  |  |  |
|  |  |  |  |  |  |
| **DATA COLLECTION AND STUDY APPRAISAL** | YES | PROB YES | PROB NO | NO | NO INFORMATION |
| Were efforts made to minimise error in data collection? | X |  |  |  |  |
| Were sufficient study characteristics available for both review authors and readers to be able to interpret the results? | X |  |  |  |  |
| Were all relevant study results collected for use in the synthesis? | X |  |  |  |  |
| Was risk of bias (or methodological quality) formally assessed using appropriate criteria? | X |  |  |  |  |
| Were efforts made to minimise error in risk of bias assessment? | X |  |  |  |  |
|  |  |  |  |  |  |
| **SYNTHESIS AND FINDINGS** | YES | PROB YES | PROB NO | NO | NO INFO |
| Did the synthesis include all studies that it should? |  | X |  |  |  |
| Were all pre-defined analyses reported or departures explained? | X |  |  |  |  |
| Was the synthesis appropriate given the nature and similarity in the research questions, study designs and outcomes across included studies? | X |  |  |  |  |
| Was between-study variation (heterogeneity) minimal or addressed in the synthesis? | X |  |  |  |  |
| Were the findings robust, e.g. as demonstrated through funnel plot or sensitivity analyses? | X |  |  |  |  |
| Were biases in primary studies minimal or addressed in the synthesis? | X |  |  |  |  |
|  |  |  |  |  |  |
| **RISK OF BIAS IN THE REVIEW** | YES | PROB YES | PROB NO | NO | NO INFO |
| Did the interpretation of findings address all of the concerns identified in Domains 1 to 4? | X |  |  |  |  |
| Was the relevance of identified studies to the review's research question appropriately considered? | X |  |  |  |  |
| Did the reviewers avoid emphasizing results on the basis of their statistical significance? | X |  |  |  |  |

**Le Brocque et al. 2020.**

|  | YES | NO | CANT TELL | COMMENTS |
| --- | --- | --- | --- | --- |
| **Are there clear research questions?** | X |  |  |  |
| **Do the collected data allow to address the research questions?** | X |  |  |  |
| Is the sampling strategy relevant to address the research question? | X |  |  |  |
| Is the sample representative of the target population? | X |  |  |  |
| Are the measurements appropriate? | X |  |  |  |
| Is the risk of nonresponse bias low? |  | X |  | “Data on 194 nonparticipants from one hospital are unfortunately unavailable due to staff error in early recruitment  procedures. Unfortunately, due to privacy regulations, we were unable to collect any medical or demographic data on those who  did not consent to participating in the study.” |
| Is the statistical analysis appropriate to answer the research question? | X |  |  |  |

**Neville et al. 2018.**

|  | YES | NO | CANT TELL | COMMENTS |
| --- | --- | --- | --- | --- |
| **Are there clear research questions?** | X |  |  |  |
| **Do the collected data allow to address the research questions?** | X |  |  |  |
| Is the sampling strategy relevant to address the research question? | X |  |  |  |
| Is the sample representative of the target population? | X |  |  |  |
| Are the measurements appropriate? | X |  |  |  |
| Is the risk of nonresponse bias low? |  |  | X | Paper did not address non-response bias but stated that there were non-participants. Did not elaborate or provide details on the characteristics of the participants nor address the risk of nonresponse bias. |
| Is the statistical analysis appropriate to answer the research question? | X |  |  |  |

**EGBERTS ET AL. 2020.**

|  | YES | NO | CANT TELL | COMMENTS |
| --- | --- | --- | --- | --- |
| **Are there clear research questions?** | X |  |  |  |
| **Do the collected data allow to address the research questions?** | X |  |  |  |
| Is the sampling strategy relevant to address the research question? | X |  |  |  |
| Is the sample representative of the target population? | X |  |  |  |
| Are the measurements appropriate? | X |  |  |  |
| Is the risk of nonresponse bias low? | X |  |  |  |
| Is the statistical analysis appropriate to answer the research question? | X |  |  |  |

**Carmassi et al. 2018.**

|  | YES | NO | CANT TELL | COMMENTS |
| --- | --- | --- | --- | --- |
| **Are there clear research questions?** | X |  |  |  |
| **Do the collected data allow to address the research questions?** | X |  |  |  |
| Is the sampling strategy relevant to address the research question? | X |  |  |  |
| Is the sample representative of the target population? | X |  |  |  |
| Are the measurements appropriate? | X |  |  |  |
| Is the risk of nonresponse bias low? |  |  | X | Paper mentions number of couples invited, number of participants, and reasons for dropout. However, no explicit overall response rate mentioned or exploration of characteristics of non-respondents. |
| Is the statistical analysis appropriate to answer the research question? | X |  |  |  |
