# Supplementary material for: The factors associated with paediatric medical post-traumatic stress: A systematic review
Source: J Health Psychol. 2024 Sep 30;30(11):2860–80. doi: 10.1177/13591053241272214 (PMC12433540; doi:10.1177/13591053241272214)
Supplement: sj-docx-2-hpq-10.1177_13591053241272214 – Supplemental material for The factors associated with paediatric medical post-traumatic stress: A systematic review [file sj-docx-2-hpq-10.1177_13591053241272214.docx]

Search Terms

Pubmed: ("medical-related posttraumatic stress" OR "medical traumatic stress" OR "PTSD" OR "Posttraumatic stress symptoms" OR "Medical-related post-traumatic stress") AND ("chronic disease" OR "chronic condition" OR "chronic") AND ("parents" OR "caregivers" OR "maternal" OR "paternal" OR "Parental") AND ("children" OR "pediatric" OR "adolescents") NOT "injuries" NOT "abuse-related injuries" NOT "Abuse")

Medline: ((("medical-related posttraumatic stress") OR ("medical traumatic stress") OR ("PTSD") OR ("Posttraumatic stress symptoms") OR ("Medical-related post-traumatic stress")) AND (("chronic disease") OR ("chronic condition") OR ("chronic")) AND (("parents") OR ("caregivers") OR ("maternal") OR ("paternal") OR ("Parental")) AND (("children") OR ("pediatric") OR ("adolescents"))) NOT ("injuries") NOT ("abuse-related injuries") NOT ("Abuse")

Scopus: ( TITLE-ABS-KEY ( "medical-related posttraumatic stress" ) OR TITLE-ABS-KEY ( "medical traumatic stress" ) OR TITLE-ABS-KEY ( "PTSD" ) OR TITLE-ABS-KEY ( "Posttraumatic stress symptoms" ) OR TITLE-ABS-KEY ( "Medical-related post-traumatic stress" ) ) AND ( TITLE-ABS-KEY ( "chronic disease" ) OR TITLE-ABS-KEY ( "chronic condition" ) OR TITLE-ABS-KEY ( "chronic" ) ) AND ( TITLE-ABS-KEY ( "parents" ) OR TITLE-ABS-KEY ( "caregivers" ) OR TITLE-ABS-KEY ( "maternal" ) OR TITLE-ABS-KEY ( "paternal" ) OR TITLE-ABS-KEY ( "Parental" ) ) AND ( TITLE-ABS-KEY ( "children" ) OR TITLE-ABS-KEY ( "pediatric" ) OR TITLE-ABS-KEY ( "adolescents" ) ) AND NOT TITLE-ABS-KEY ( "injuries" ) AND NOT TITLE-ABS-KEY ( "abuse-related injuries" ) AND NOT TITLE-ABS-KEY ( "Abuse" )
